# Transvenous vagus nerve stimulation does not modulate the innate immune response during experimental human endotoxemia: a randomized controlled study

**DOI:** 10.1186/s13075-015-0667-5

**Published:** 2015-06-07

**Authors:** Matthijs Kox, Lucas T. van Eijk, Tim Verhaak, Tim Frenzel, Harmke D. Kiers, Jelle Gerretsen, Johannes G. van der Hoeven, Lilian Kornet, Avram Scheiner, Peter Pickkers

**Affiliations:** Department of Intensive Care Medicine, Radboud University Medical Center, Radboud Center for Infectious Diseases (RCI), Geert Grooteplein 10, 6500 HB Nijmegen, The Netherlands; Medtronic Inc., Endepolsdomein 5, 6229 GW Maastricht, The Netherlands; Medtronic Inc., 8200 Coral Sea St NE, Mounds View, MN 55112 USA

## Abstract

**Introduction:**

Vagus nerve stimulation (VNS) exerts beneficial anti-inflammatory effects in various animal models of inflammation, including collagen-induced arthritis, and is implicated in representing a novel therapy for rheumatoid arthritis. However, evidence of anti-inflammatory effects of VNS in humans is very scarce. Transvenous VNS (tVNS) is a newly developed and less invasive method to stimulate the vagus nerve. In the present study, we determined whether tVNS is a feasible and safe procedure and investigated its putative anti-inflammatory effects during experimental human endotoxemia.

**Methods:**

We performed a randomized double-blind sham-controlled study in healthy male volunteers. A stimulation catheter was inserted in the left internal jugular vein at spinal level C5–C7, adjacent to the vagus nerve. In the tVNS group (n = 10), stimulation was continuously performed for 30 minutes (0–10 V, 1 ms, 20 Hz), starting 10 minutes before intravenous administration of 2 ng kg^−1^*Escherichia coli* lipopolysaccharide (LPS). Sham-instrumented subjects (n = 10) received no electrical stimulation.

**Results:**

No serious adverse events occurred throughout the study. In the tVNS group, stimulation of the vagus nerve was achieved as indicated by laryngeal vibration. Endotoxemia resulted in fever, flu-like symptoms, and hemodynamic changes that were unaffected by tVNS. Furthermore, plasma levels of inflammatory cytokines increased sharply during endotoxemia, but responses were similar between groups. Finally, cytokine production by leukocytes stimulated with LPS *ex vivo*, as well as neutrophil phagocytosis capacity, were not influenced by tVNS.

**Conclusions:**

tVNS is feasible and safe, but does not modulate the innate immune response in humans in vivo during experimental human endotoxemia.

**Trial registration:**

Clinicaltrials.gov NCT01944228. Registered 12 September 2013.

## Introduction

Inflammatory cytokines are pivotal in the pathogenesis of rheumatoid arthritis (RA) [1]. Biologics that antagonize these cytokines (or their receptors) are very effective and have revolutionized the treatment of RA [2]. However, they are very expensive and can have serious side effects [3]. Therefore, innovative non-pharmacological anti-inflammatory therapies for RA are highly warranted.

More than a decade ago, the group of Kevin Tracey discovered the so-called “cholinergic anti-inflammatory pathway” [4]. In rats, it was demonstrated that electrical stimulation of the efferent vagus nerve (vagus nerve stimulation (VNS)) inhibits the systemic inflammatory response to endotoxin (lipopolysaccharide (LPS)) administration through release of the vagal neurotransmitter acetylcholine [4]. Since these initial discoveries, multiple studies in rodents have shown that VNS limits the immune response during endotoxemia [5, 6], and exerts beneficial effects in animal models of live bacterial sepsis, trauma, ischemia–reperfusion injury, and hemorrhagic shock [7–10]. Moreover, VNS as well as pharmacological stimulation of the cholinergic anti-inflammatory pathway attenuates collagen-induced arthritis in mice and rats [11–13]. As such, VNS has been implicated as a novel treatment modality for RA [14, 15].

In humans, VNS is FDA-approved for the treatment of epilepsy and depression using an implantable VNS system including a cuff electrode wrapped around the left vagus nerve [16, 17]. However, until now there is very little evidence for anti-inflammatory effects of VNS in humans [18, 19], and no standardized models of inflammation have been exploited to investigate these putative anti-inflammatory effects. Furthermore, the methods to apply VNS that have been used so far require invasive surgery [18, 19]. Recently, a novel method to apply VNS, transvenous VNS (tVNS), has been developed [20, 21]. tVNS employs electrodes placed in the internal jugular vein (IJV) at spinal level C5–C7, where the IJV runs adjacent to the vagus nerve, representing a less invasive way to stimulate the vagus nerve.

The anti-inflammatory potential of biologics that are currently used for the treatment of RA was first established during human endotoxemia [22, 23], a standardized, controlled model of systemic inflammation in humans in vivo [24–26]. This illustrates the relevance of the model to test novel therapies for RA. In the present study, we determined whether tVNS is a feasible and safe procedure and investigated if it exerts anti-inflammatory effects in healthy male volunteers during experimental human endotoxemia.

## Methods

### Subjects and randomization

After approval from the ethics committee of the Radboud University medical center, 20 healthy young non-smoking male volunteers gave written informed consent to participate in this randomized, double-blind, sham-controlled study (Clinicaltrials.gov NCT01944228). All experiments were conducted in accordance with the declaration of Helsinki. Screening took place within 14 days before the experiment and showed no abnormalities in physical examination, electrocardiography, and routine laboratory tests. Subjects taking prescription medication or experiencing clinically significant illness within 14 days before the endotoxemia experiment day were not allowed to participate. The day before the experiment, subjects were not allowed alcohol and caffeine intake and 10 hours before experimental endotoxemia they refrained from ingesting food. Subjects were randomly assigned in pairs to receive either tVNS (n = 10) or sham tVNS (n = 10). Randomization was performed by unblinded personnel through numbered envelopes.

### Study outline

**Fig. 1:**
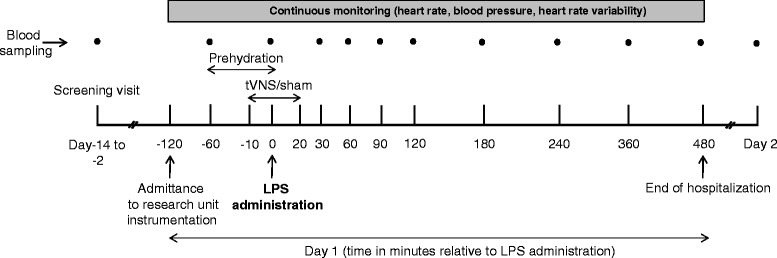
Schematic overview of the study procedures. *LPS* lipopolysaccharide, *tVNS* transvenous vagus nerve stimulation

Figure 1 provides a schematic overview of the study procedures. Briefly, on the experimental endotoxemia day (day 1), after a re-check of exclusion criteria, a cannula was placed in the antecubital vein of the non-dominant arm, and the radial artery of the same arm was cannulated under local anesthesia (lidocaine HCl 20 mg mL^−1^) using a 20-gauge arterial catheter for continuous monitoring of blood pressure and sampling of blood. Subjects received 1.5 L of 2.5 % glucose/0.45 % saline solution for 1 hour (prehydration) before LPS administration, followed by 150 mL h^−1^ until 6 hours after LPS infusion and 75 mL h^−1^ until the end of the experiment (8 hours after LPS administration). Heart rate was measured continuously using a 3-lead electrocardiogram. Every 30 minutes, temperature was measured using an infrared tympanic thermometer (First-Temp Genius, Sherwood Medical, Crawley/Sussex, UK), and symptoms (nausea, headache, muscle ache, back pain, and shivering) were scored (ranging from 0 (symptom not present) to 5 (worst ever experienced), in case of vomiting 3 points were added), forming an arbitrary total symptom score with a maximum of 28 points. Purified LPS (US Standard Reference Endotoxin Escherichia Coli O:113) obtained from the Pharmaceutical Development Section of the National Institutes of Health (Bethesda, MD, USA), supplied as a lyophilized powder, was reconstituted in 5 mL saline 0.9 % for injection and vortex-mixed for at least 20 minutes after reconstitution. The LPS solution was administered as an intravenous bolus injection at a dose of 2 ng kg^−1^ body weight over 1 minute at T = 0 hours. tVNS/sham stimulation (detailed below) started 10 minutes before LPS administration and was stopped 20 minutes after LPS administration.

### tVNS/sham stimulation procedure

Subjects were prepared for catheter insertion by standard methods of disinfecting an IJV entry site. After local anesthesia (lidocaine HCL 20 mg mL^−1^) the left IJV was cannulated under ultrasound guidance, using a two-lumen central venous catheterization set with blue FlexTip® (Arrow international, Inc., Reading, PA, USA). A stimulation catheter with eight electrodes on a circular distal loop (Achieve Medtronic model 990063-20, Medtronic, Heerlen, The Netherlands) was connected to the electrode switch box (Medtronic model 19038) with a catheter cable (Medtronic model 990066). A N’Vision Clinical Programmer (Medtronic model 8840) was used to program the external neurostimulator (Medtronic model 37022), which was connected to the electrode switch box via the Trialing System cable (Medtronic model 19039). The stimulation catheter was positioned at the C5–C7 spinal level, which was shown to result in vagus nerve stimulation in previous clinical studies [20, 21]. The position of the distal loop of the stimulation catheter adjacent to the vessel wall was confirmed by ultrasound. During positioning of the catheter and (sham) stimulation, blinded study personnel left the room. The subjects were blinded by being told that they could either feel the stimulation, but not necessarily so, and that the sham group would also receive stimulation but with a “non-therapeutic pattern”. Determination of the correct electrode pair for stimulation in the tVNS group was attained by first switching between different electrode pair combinations with the stimulator turned on until laryngeal vibration was felt, a reliable indicator of actual stimulation of the vagus nerve [20, 21, 27–29]. Subsequently, the voltage thresholds at which laryngeal vibrations were still felt were determined for the respective electrode pair and the adjacent electrode pair. The electrode pair with the lowest laryngeal threshold was selected for stimulation. Next, the voltage was raised to determine the maximum voltage output at which the subject still felt comfortable. In the tVNS group, stimulation was continuously applied during 30 minutes using the following stimulation parameters: 2–10 V amplitude (see Table 1 for laryngeal thresholds and stimulation voltages used in each subject), 1 ms pulse width, 20 Hz frequency, consistent with the settings used in preclinical and clinical studies and well within safety margins obtained from these studies [6, 20, 21].

**Table 1:** Stimulation parameters in the transvenous vagus nerve stimulation group

	Laryngeal threshold (V)	Start (V)	End (V)
Subject 1	2.3	5.5	8.0
Subject 2	3.0	4.5	6.1
Subject 3	2.0	7.5	9.5
Subject 4	3.0	7.9	8.5
Subject 5	1.0	2.0	2.4
Subject 6	1.0	2.0	3.0
Subject 7	1.5	2.5	3.8
Subject 8	2.0	4.5	8.1
Subject 9	1.0	6.4	8.8
Subject 10	2.0	8.0	10.0

*Laryngeal threshold* the lowest voltage at which laryngeal vibrations were felt. *Start* voltage used at the start of the 30-minute stimulation period. *End* voltage used at the end of the 30-minute stimulation period. Start and end indicate maximum voltages at which the subject still felt comfortable

Due to anticipated stimulation-related side effects, the voltage was gradually increased during the stimulation period, ensuring it did not result in discomfort for the subjects. In the sham group, the exact same procedures were performed, but the stimulator was not switched on. To be consistent in the procedures (for the blinded subjects) and to exclude a possible stimulation effect we verified that no vagus nerve stimulation occurred as confirmed by the absence of laryngeal vibration. Subjects were told to report any discomfort. If the subject felt pain or discomfort, the stimulation amplitude was reduced until the sensation was eliminated. Catheter location, stimulation parameters, laryngeal vibration, and any side effects were noted on a data collection form that was kept in a separate folder until unblinding of the study.

### Plasma cytokine measurements

Ethylenediaminetetraacetic acid (EDTA) anti-coagulated blood was immediately centrifuged at 2000 g for 10 minutes at 4 °C after which plasma was stored at –80 °C until analysis. Cytokine concentrations were determined using a Luminex Assay according to the manufacturer’s instructions (Milliplex, Millipore, Billerica, MA, USA). Lower detection limit was 3.2 pg mL^−1^ for all cytokines.

### Leukocyte counts

Analysis of leukocyte counts was performed in EDTA anticoagulated blood using routine analysis methods also used for patient samples (flow cytometric analysis on a Sysmex XE-5000 (Etten-Leur, The Netherlands)).

### Ex vivo whole blood stimulation

Leukocyte cytokine production capacity was determined before (sham) tVNS (time-point T = –1 hours), during (sham) tVNS immediately before LPS administration (time-point T = 0), and at 2, 4, and 8 hours after LPS administration. Cytokine production capacity was assessed by challenging whole blood from the subjects with LPS ex vivo using an in-house developed system with pre-filled tubes described in detail elsewhere [30]. Briefly, 0.5 mL of blood was added to tubes pre-filled with 2 mL culture medium or 2 mL culture medium supplemented with 12.5 ng mL^−1^ LPS (end concentration of LPS 10 ng mL^−1^). Cultures were incubated at 37 °C for 24 hours, centrifuged, and supernatants were stored at –8 °C until analysis. Concentrations of tumor necrosis factor (TNF)-α and interleukin (IL)-6 were determined using enzyme-linked immunosorbent assays according to the manufacturer’s instructions (Duosets, R&D systems, Minneapolis, MN, USA). Cytokine concentrations in supernatants obtained from negative control tubes were subtracted from concentrations in supernatants obtained from LPS tubes.

### Neutrophil phagocytosis assay

Phagocytosis capacity was determined at the same time-points as ex vivo whole blood stimulation (see previous paragraph) using the pHrodo Red *Escherichia coli* BioParticles Phagocytosis Kit for Flow Cytometry according to the manufacturer’s instructions (Life Technologies, Bleiswijk, The Netherlands). Briefly, 100 μL lithium-heparin anticoagulated blood was incubated with pHrodo Red *E. coli* BioParticles at 37 °C for 15 minutes after which erythrocytes were lysed. After several washing steps, leukocytes were stored in 1 % paraformadelhyde solution until flow cytometric analysis on a Beckman Coulter Cytomics FC500 flow cytometer (Beckman Coulter, Galway, Ireland). Neutrophils were gated using forward side-scatter characteristics. Percentage of pHrodo-positive neutrophils (phycoerythrin channel) were determined using a negative control (blood incubated with pHrodo Red *E. coli* BioParticles on ice). Furthermore, mean fluorescence intensity (MFI) of the pHrodo-positive neutrophil population was determined and the phagocytic index was calculated using the formula: percentage pHrodo positive neutrophils × MFI of pHrodo-positive neutrophils.

### Statistical analyses

Data are expressed as mean and standard error of the mean (SEM) or median with interquartile ranges (IQR) of the 25^th^ and 75^th^ percentile based on distribution (determined using Shapiro-Wilk tests). Statistical tests were used as appropriate based on distribution and are indicated in the text and table and figure legends. A *p* value < 0.05 was considered statistically significant. Statistical analyses were performed using Graphpad Prism 5.03 (Graphpad Software, San Diego, CA, USA).

## Results

### Demographic characteristics and safety

There were no significant differences in baseline characteristics between the study groups (Table 2). No serious adverse events occurred during the conduct of the study.

**Table 2:** Demographic characteristics

	Sham (n = 10)	tVNS (n = 10)	*p*-value
Age (years)	24 (22–26)	26 (23–27)	0.32
Height (cm)	185 (177–188)	184 (181–186)	0.97
Weight (kg)	76 (69–86)	80 (68–81)	1.00
BMI (kg m^-2^)	22.2 (20.6–25.5)	22.0 (20.1–25.0)	0.91

Data are presented as median (interquartile range). *p*-values calculated using Mann-Whitney *U*-test. *BMI* body mass index, *tVNS* transvenous vagus nerve stimulation

### tVNS/sham stimulation procedure

In all 20 subjects, placement of the stimulation electrode was successful and uneventful. In all 10 subjects of the tVNS group, laryngeal vibration was felt, indicating stimulation of vagal fibers (Table 1). Placement of the stimulation catheter and determination of the correct electrode pair and stimulation voltage took 40 minutes on average. Side effects of stimulation during assessment of the correct stimulation voltage included an uncomfortable feeling and/or difficulty in swallowing, throat spasms, neck muscle contractions, and changes in vocal sound. In case of side effects, the voltage was lowered. Stimulation was well-tolerated. In all 10 subjects it was possible to raise the voltage during the stimulation period, indicating that the subjects accommodated to the stimulation (Table 1).

### Hemodynamic parameters, temperature, and symptoms

**Fig. 2:**
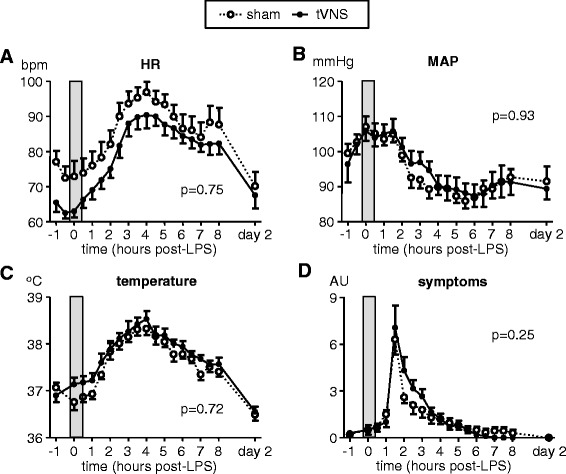
Hemodynamic parameters, temperature, and symptoms. **a** Heart rate (*HR*). **b** Mean arterial pressure (*MAP*). **c** Temperature. **d** Score of self-reported symptoms. Data are expressed as mean and SEM of 10 subjects per group. The *gray box* indicates the period in which the (sham) stimulation took place. Within-group changes over time were all highly significant (*p* < 0.0001 for all parameters in both groups, calculated using repeated measures one-way ANOVA). *p*-values depicted indicate differences between groups over time calculated using repeated measures two-way ANOVA (interaction term). *AU* arbitrary units, *LPS* lipopolysaccharide, *tVNS* transvenous vagus nerve stimulation

LPS administration resulted in a typical increase in body temperature, flu-like symptoms, and heart rate, as well as a decrease in mean arterial pressure (Fig. 2), with no differences between the tVNS and sham groups.

### Plasma cytokines and leukocyte numbers

**Fig. 3:**
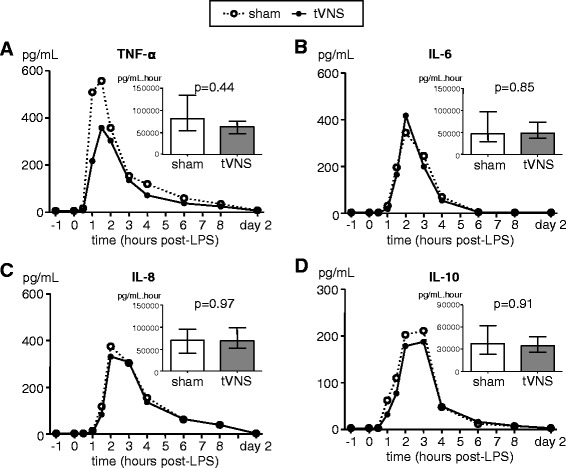
Plasma concentrations of inflammatory cytokines. **a** Tumor necrosis factor (*TNF*)-α. **b** Interleukin (*IL*)-6. **c** IL-8 (**d**) IL-10. Data are expressed as medians of 10 subjects per group. Inserted bar graphs depict median and interquartile range of area under curve of cytokine time courses. Within-group changes over time were all highly significant (*p* < 0.0001 for all cytokines in both groups, calculated using Friedman tests). *p*-values depicted were calculated using Mann-Whitney U-tests. *LPS* lipopolysaccharide, *tVNS* transvenous vagus nerve stimulation

Administration of LPS resulted in a sharp increase in plasma levels of the pro-inflammatory cytokines TNF-α, IL-6, and IL-8 as well as the anti-inflammatory cytokine IL-10 (Fig. 3). No differences in plasma levels of these cytokines were observed between the tVNS and sham groups.

After LPS administration, leukocyte numbers showed a typical initial decrease (tVNS, from 4.9 ± 1.1 × 10^9^ L^−1^ at T = 0 hours to 3.7 ± 1.2 × 10^9^ L^−1^ at T = 1 hours; sham, from 5.5 ± 1.4 × 10^9^ L^−1^ at T = 0 hours to 3.4 ± 1.9 × 10^9^ L^−1^ at T = 1 hours), followed by leukocytosis, peaking at T = 8 hours (tVNS, 10.2 ± 2.4 × 10^9^ L^−1^; sham, 11.6 ± 2.2 × 10^9^ L^−1^). At day 2, leukocyte numbers were normalized (tVNS, 4.9 ± 1.0 × 10^9^ L^−1^; sham, 6.0 ± 0.8 × 10^9^ L^−1^). There were no differences between the tVNS and sham groups (*p* = 0.51, two-way ANOVA interaction term).

### Ex vivo whole blood stimulation and neutrophil phagocytosis capacity

**Fig. 4:**
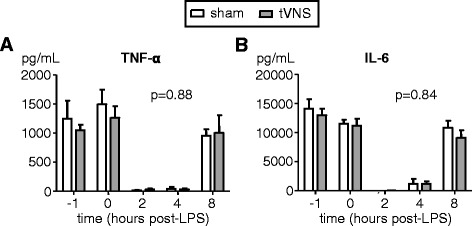
Cytokine production of leukocytes stimulated ex vivo with lipopolysaccharide (*LPS*). **a** Tumor necrosis factor (*TNF*)-α. **b** Interleukin (*IL*)-6. Data expressed as mean and SEM of 10 subjects per group. Within-group changes over time were all highly significant (*p* < 0.0001 for all parameters in both groups, calculated using repeated measures one-way ANOVA). *p*-values depicted indicate differences between groups over time calculated using repeated measures two-way ANOVA (interaction term). *tVNS* transvenous vagus nerve stimulation

We evaluated the effects of tVNS on cytokine production of leukocytes stimulated with LPS ex vivo and neutrophil phagocytosis, because previous studies have indicated that VNS results in altered leukocyte function [31–34]. LPS administration resulted in a profound transient reduction in production of TNF-α and IL-6 by leukocytes stimulated with LPS ex vivo in both groups, which normalized after 8 hours (Fig. 4). There were no differences in production of TNF-α or IL-6 between the tVNS and sham groups over time (two-way ANOVA interaction term indicated in Fig. 4) or at any of the separate time-points (unpaired Student t-tests).

**Fig. 5:**
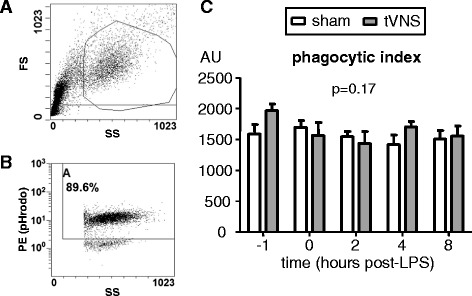
Neutrophil phagocytosis capacity. **a** Representative example of gating of neutrophils in the leukocyte population using forward side-scatter characteristics. **b** Representative example of gating of phRodo positive-neutrophils (phycoerythrin (*PE*) channel) within the neutrophil gate. **c** Phagocytic index calculated using the formula: pHrodo positive neutrophils × MFI of pHrodo-positive neutrophils. Data in panel c are expressed as mean and SEM of 10 subjects per group. Within-group changes over time were not significant (*p* = 0.38 and *p* = 0.14 for the sham and tVNS groups, respectively, calculated using repeated measures one-way ANOVA). *p*-values depicted indicate differences between groups over time calculated using repeated measures two-way ANOVA (interaction term). *AU* arbitrary units, *FS* forward scatter, *LPS* lipopolysaccharide, *SS* side scatter, *tVNS* transvenous vagus nerve stimulation

LPS administration did not result in altered phagocytosis capacity of neutrophils, reflected by the percentage of phagocytosing neutrophils, fluorescence intensity of phagocytosing neutrophils, and phagocytic index within both groups (the latter parameter is depicted in Fig. 5). Furthermore, there were no differences in phagocytosis capacity between the tVNS and sham groups over time (two-way ANOVA interaction term indicated in Fig. 5) or at any of the separate time-points (unpaired Student t-tests).

## Discussion

VNS has been shown to exert potent anti-inflammatory effects in animal studies and is implicated in representing a putative treatment modality for RA. Nevertheless, until now human data are largely lacking. In the present study we demonstrate that tVNS is a feasible and well-tolerated procedure in young healthy male volunteers. However, tVNS neither affected plasma cytokine levels and flu-like symptoms, nor fever and hemodynamic parameters during experimental human endotoxemia. In addition, no effects of tVNS on cytokine production by leukocytes stimulated with LPS ex vivo or neutrophil phagocytosis capacity were observed. To the best of our knowledge, we are the first to investigate the effects of VNS in a standardized well-controlled model of systemic inflammation in humans in vivo that is widely used to investigate immunomodulatory therapies [35]. Therefore, although negative, our results are relevant, especially because more than 60 clinical studies are currently underway investigating VNS for the treatment of RA and various other disorders, including Crohn’s disease, stroke, and heart failure [36].

The finding that tVNS does not result in anti-inflammatory effects are in contrast to a range of animal studies where potent anti-inflammatory effects of VNS were found [5–10]. This discrepancy might be explained by several reasons. First, species differences could play a role in both the response to endotoxin administration [37] and the extent of the anti-inflammatory effects of VNS. Second, one might argue that the transvenous approach did not result in adequate/appropriate stimulation of the vagus nerve. This possibility appears highly unlikely, since clear laryngeal vibration was observed in all subjects of the tVNS group and laryngeal side effects have shown to be a reliable indication of stimulation of the vagus nerve using the cuff electrode VNS system [27–29]. Heart rate variability (HRV) analysis, a widely used method to determine vagus nerve activity, is not suited for assessing VNS in our setting as it merely reflects cardiac vagal effects, which are predominantly mediated by the right vagus nerve. Analogous to virtually all animal and human VNS studies, we stimulated the left vagus nerve to minimize cardiac side effects and to increase outflow to the spleen, which is implicated in mediating the anti-inflammatory effects of VNS [38]. In agreement, previous studies have demonstrated that stimulation of the left vagus nerve using the cuff electrode system does not result in increased vagal HRV parameters [39–42]. Also, previous animal work has shown that the anti-inflammatory effects of stimulation of the left vagus nerve are independent from cardiac vagal effects [6]. A third possible explanation could be that the stimulation parameters employed in this study might not be adequate to produce anti-inflammatory effects. However, a murine endotoxemia study demonstrated that anti-inflammatory effects of VNS are similar using voltages as low as 1 V as well as various pulse widths and frequencies ranging from 0.5 to 2 ms, and from 5 to 30 Hz, respectively [6]. We used the highest tolerable voltage per subject to maximize stimulation of the nerve. Furthermore, the 30-minute duration of stimulation used in our study appears to be adequate, as many animal studies used 10–20 minutes [4, 5, 7–10, 38, 43] and anti-inflammatory effects were shown to be similar using stimulation durations ranging from 0.5 to 20 minutes [6].

As mentioned before, human studies into the anti-inflammatory effects of VNS are very scarce, related to the technical difficulties in stimulating the vagus nerve and large variation in the inflammatory response in the clinical setting. Furthermore, the interpretation of these studies is hampered by the fact that in all but one VNS study no inflammatory trigger was present. As such, circulating cytokine levels in the study subjects (patients suffering from epilepsy or depression) were in many cases undetectable or otherwise very low [39, 44, 45]. Another major difference between these studies and ours is that, previously, effects of chronic VNS were investigated (3–7 months, during which patients received constant stimulation). The results of the studies in which no inflammatory trigger was present are conflicting, with two studies demonstrating no change in plasma cytokine levels 3 months [39] or 7 months [45] after VNS, while another found that plasma cytokine levels were actually increased after 3 months of VNS [44]. One study investigated the effects of VNS for 3 weeks and 6 months on cytokine production by monocytes ex vivo stimulated with LPS. Out of five measured cytokines at two different time-points, only ex vivo production of IL-8 was significantly lower 6 months after implantation of the stimulator device [18]. Only one study assessed the effects of vagal stimulation in a clinical setting known to be associated with an inflammatory response, namely coronary artery bypass graft surgery [19]. In this study, epicardial vagal ganglionated plexus stimulation was applied using a temporary wire electrode placed into the vagal fat pad on the right ventricle, which resulted in anti-inflammatory effects [19]. However, the VNS approach used in this particular study is very different from all other studies. As such, other effects, such as direct effects on the right ventricle, may play a role in the observed results. Of interest, very recently a pilot study was carried out in which eight RA patients were implanted with the cuff electrode VNS system (only published in abstract form [46]). In these patients, VNS was well tolerated but no significant reduction in C-reactive protein (CRP), Disease Activity Score (DAS)28-CRP, or Health Assessment Questionnaire Disability Index (HAQ-DI) was observed 42 days after implantation and start of VNS.

Along with our main endpoint, i.e., plasma cytokine levels, several other inflammatory parameters that have been demonstrated or suggested to be affected by VNS were evaluated. For instance, it has been proposed that VNS “reprograms” passing leukocytes in the spleen, resulting in decreased cytokine production capacity and recruitment to sites of inflammation by these cells [31, 33, 34]. This was the rationale for us and others [18] to examine cytokine production of leukocytes stimulated with LPS ex vivo. Typically, and similar to previous studies [25, 47–49], we found a transient reduction in leukocyte cytokine production after LPS administration in vivo, a phenomenon known as endotoxin tolerance. However, no effects of tVNS were observed at any of the time-points studied. This indicates that tVNS neither exerts immediate effects on leukocyte cytokine production capacity, nor does it affect the development of endotoxin tolerance in these cells. We also evaluated neutrophil phagocytosis capacity, because VNS has been reported to enhance phagocytosis in mice [32], possibly also via reprogramming leukocytes in vagus-innervated organs. To the best of our knowledge, this is the first time that neutrophil phagocytosis has been evaluated in the human endotoxemia model. We did not find any effects of LPS administration on the capacity of neutrophils to phagocytose *E. coli* bioparticles. Therefore, it appears that the profound endotoxin tolerance observed in leukocytes after LPS administration in vivo in terms of cytokine production does not apply to phagocytic function. This is in agreement with an in vitro study showing that incubation of whole blood with LPS does result in severely impaired monocytic cytokine production capacity upon restimulation with LPS, while phagocytosis remains unaffected [50]. Again, in line with all our other results, no effects of tVNS on neutrophil phagocytosis were observed.

## Conclusions

tVNS is feasible and safe, but does not influence the systemic inflammatory response in vivo in humans during experimental endotoxemia. These results indicate that short-term tVNS does not modulate the innate immune response in humans, and that the therapeutic benefit in RA patients may be limited.

## Abbreviations

ANOVA: Analysis of variance
CRP: C-reactive protein
DAS: Disease activity score
EDTA: Ethylenediaminetetraacetic acid
HAQ-DI: Health Assessment Questionnaire Disability Index
HRV: Heart rate variability
IJV: Internal jugular vein
IL: Interleukin
IQR: Interquartile range
LPS: Lipopolysaccharide
MFI: Mean fluorescence intensity
RA: Rheumatoid arthritis
SEM: Standard error of the mean
TNF: Tumor necrosis factor
tVNS: Transvenous vagus nerve stimulation
VNS: Vagus nerve stimulation
